# Modeling of a Symmetric Five-Bar Displacement Amplification Compliant Mechanism for Energy Harvesting

**DOI:** 10.3390/s21041095

**Published:** 2021-02-05

**Authors:** Moataz M. Elsisy, Mustafa H. Arafa, Chahinaz A. Saleh, Yasser H. Anis

**Affiliations:** 1 Mechanical Design and Production Department, Faculty of Engineering, Cairo University, Giza 12613, Egypt; mme41@pitt.edu (M.M.E.); chahinaz@eng.cu.edu.eg (C.A.S.); 2 Mechanical Engineering Department, American University in Cairo, New Cairo 11835, Egypt; mharafa@aucegypt.edu

**Keywords:** displacement amplification, compliant mechanism, castigliano’s theorem, energy harvesting

## Abstract

This paper presents an analytical model to determine a closed form mathematical representation for the output displacement of a displacement amplification compliant mechanism used for energy harvesting. A symmetric five-bar compliant mechanism with right-circular and corner-filleted flexure hinges was mathematically modeled and its displacement was determined using the Castigliano energy theorem. The stresses within the flexure joints, the weakest points in the mechanism body, were calculated. The mathematical model expresses both the displacement amplification and the stresses as functions of the design parameters and the load caused by the harvester. The developed model can be used to optimize the mechanism dimensions for maximum harvested power, while minimizing its structural stresses. The mechanism was also modeled numerically using finite element methods; both the analytical and numerical models were verified experimentally. The mathematical model of the mechanism was integrated with a model representing a piezoelectric energy harvester to calculate the open-circuit voltage. As a proof of concept, experiments were performed using an unimorph piezoelectric cantilever at low-frequency (less than 1 Hz) harmonic excitation inputs. The measured open-circuit voltage was found to be in agreement with that calculated using the proposed model, when integrated with the model representing the piezoelectric beam. The power generated by the piezoelectric harvester, equipped with the proposed displacement amplification mechanism, was more than a hundred times that without amplification.

## 1. Introduction

Interest in vibration-based energy harvesting has witnessed significant growth in recent years due to the ubiquity of mechanical vibrations as a viable source of energy for many applications. One of the main challenges, however, is that many practical sources of ambient vibration provide low-frequency, low-amplitude motion, which places limitations on the design of harvesters that would best respond to such excitation schemes to capture energy effectively. This has prompted research into maximizing the input motion by increasing the frequency range of vibration energy harvesters [1,2]. Several techniques have been suggested to increase the frequency range; thus, maximize harvested power, including natural frequency tuning [3,4,5], in addition to bandwidth widening using an array of structures, coupled oscillators, nonlinear springs, or bi-stable structures [6,7]. The excitation force and frequency may require modulation to increase the output power [8]. Mechanical modulation methods include frequency up-conversion [9,10], and excitation amplification mechanisms.

Excitation amplification mechanisms have been used, including lever mechanisms, scissor linkage mechanisms, and rack-pinion mechanisms [11,12]. Due to backlash and clearance problems associated with these systems, compliant mechanisms were proposed, including flextensional mechanisms [13], in addition to displacement amplification compliant mechanisms [14,15,16,17].

In this work, a compliant mechanism is used for amplifying the excitation displacement. Compliant mechanisms are flexible mechanisms that achieve their mobility from the flexibility of their flexure joints. They are advantaged over traditional link-joints mechanisms in their simplicity of fabrication, lighter weights, reduced wear and backlash, and high precision, which makes them favorable for use in dirty and harsh environments [18]. The proposed device can be particularly useful for harvesting energy in applications involving low-frequency, low-amplitude vibration, such as railway infrastructure, bridges, and buildings, to extract energy for operating self-powered sensors. In such environments, the excitation amplitude is usually too small to drive generators directly and hence can be amplified for greater harvested power output. Ideally, the device works when inserted in confined structural spaces in which a prevailing relative motion drives the compliant mechanism against a fixed reference surface.

When employed in the context of vibration-based energy harvesting, the objective of the compliant mechanism is to condition a given input displacement through amplifying its magnitude. This makes it easier to extract greater amounts of power from the ambient energy sources and should be performed while ensuring the longevity and structural integrity of the compliant mechanism and its constituent joints. These objectives often demand higher amplification ratios and greater output stiffness of the compliant mechanism. As these competing attributes are often conflicting, a trade-off must usually be made to come up with a compromise, which can be conducted through proper modeling and simulation tools. Compliant mechanisms have been modeled using pseudo-rigid-body models (PRBM) [14,19,20,21,22], or topology optimization methods [23,24]. The mathematical modeling of compliant mechanisms with right-circular and corner-filleted flexure joints have also been performed using Castigliano’s energy theorem [25], and the principle of virtual work [26].

**Figure 1:**
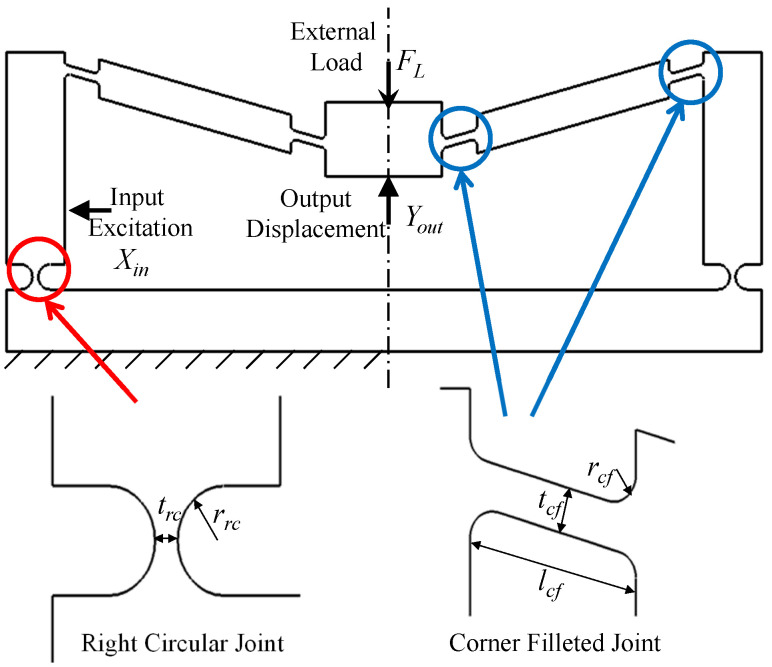
Symmetric five-bar mechanism schematic showing flexure joints dimensions.

In this paper, we present an analytical model for the symmetric five-bar displacement amplification compliant mechanism used in [14,15,27], shown in Figure 1. This mechanism is composed of links, corner-filleted flexure joints, and right-circular flexure joints, and is advantaged for its ability to provide high amplification ratios. Castigliano’s energy theorem was used to find closed-form mathematical representations of the output displacement and the normal stresses in the flexure joints. The mechanism was also modeled numerically using finite element methods; both the analytical and numerical models were verified experimentally. Preliminary results of this work were reported in [15]. The mathematical model of the mechanism was integrated with a model representing a piezoelectric energy harvester.

## 2. Displacement Amplification

**Figure 2:**
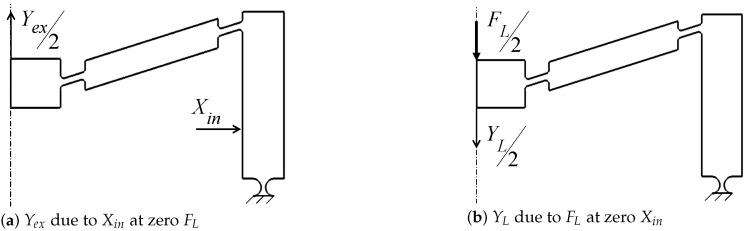
Output displacements $Y_{ex}$ and $Y_{L}$.

Subjecting the mechanism to a horizontal input displacement $X_{in}$ results in an output displacement $Y_{out}$, (see Figure 1). The ratio between $Y_{out}$ and $X_{in}$ defines the mechanism’s amplification ratio. Based on the principle of superposition, and assuming linear elastic behavior, $Y_{out}$ is the difference between two displacements, $Y_{ex}$, and $Y_{L}$, as in:

(1) $$Y_{out}=Y_{ex}-Y_{L},$$

where $Y_{ex}$ is the output displacement resulting from $X_{in}$ at zero external load $F_{L}$, while $Y_{L}$ results from an external load $F_{L}$ at zero $X_{in}$, (see Figure 2). Due to the symmetry in the mechanism topology, only half the mechanism is analyzed to reduce computational efforts and resources; thus, both $F_{L}$, and $Y_{ex}$ are halved. The displacements $X_{in}$, $Y_{out}$, $Y_{ex}$, and $Y_{L}$ are all measured from the mechanism’s static equilibrium position. The amplification ratio AR is therefore written as:

(2) $$AR=\frac{Y_{ex}-Y_{L}}{X_{in}}.$$

**Figure 3:**
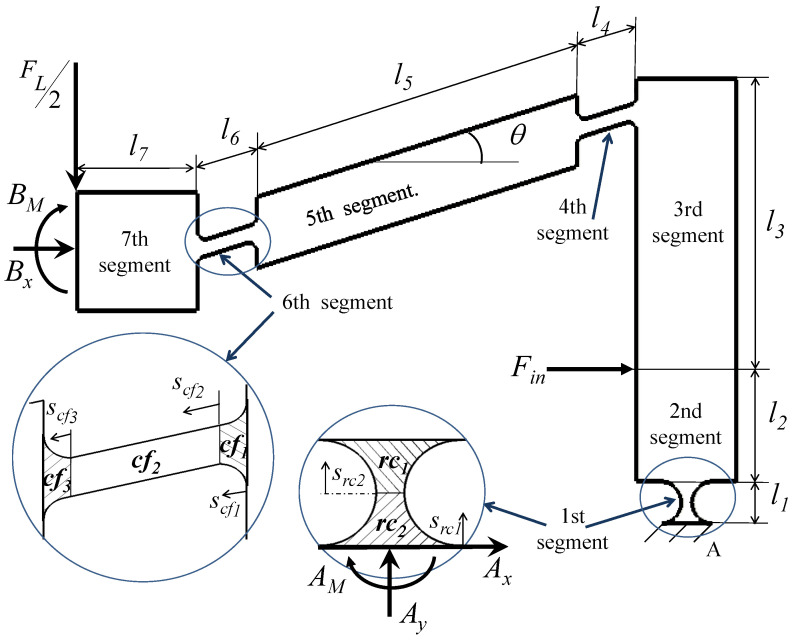
Free body diagram of the mechanism, showing dimensions, forces, and mechanism segments “*i*”.

Figure 3 represents a free body diagram of half the mechanism, divided into seven segments. $B_{M}$ and $B_{x}$ are the internal reactions due to the symmetric boundary conditions, while $A_{x}$, $A_{y}$ and $A_{M}$ are the external reactions at the fixation point “A”. $F_{in}$ is the force that produces the input displacement $X_{in}$, which can be represented using the concepts of Castigliano’s energy theorem as:

(3) $$X_{in}=\sum_{i=1}^{7}\;\int_{0}^{l_{i}}\frac{M_{i}\;\frac{d\;M_{i}}{d\;F_{in}}}{E\;I_{i}}\;ds_{i},$$

where $M_{i}$ is the internal reaction moment for each mechanism segment *i*. For every segment *i*, $s_{i}$ is the distance measured from the beginning of the segment in the counter-clockwise direction, *E* is the elastic modulus of the mechanism material, $l_{i}$ is the length of the segment, and $I_{i}$ is its second moment of area. The energy consumed in shear is neglected.

Using Castigliano’s energy theorem, the displacements $Y_{L}$ and $Y_{ex}$ can similarly be represented as:

(4) $$\begin{array}{cc} Y_{ex}= & {\left.\sum_{i=1}^{7}\int_{0}^{l_{i}}\frac{M_{i}\frac{dM_{i}}{dF_{L}}}{EI_{i}}ds_{i}\right|}_{F_{L}=0}, \end{array}$$

(5) $$\begin{array}{cc} Y_{L}= & {\left.2\sum_{i=1}^{7}\int_{0}^{l_{i}}\frac{M_{i}\frac{dM_{i}}{dF_{L}}}{EI_{i}}ds_{i}\right|}_{X_{in}=0}. \end{array}$$

In energy harvesting applications, the vibration source excites the mechanism with the displacement $X_{in}$. The resulting amplified output displacement $Y_{out}$ actuates the harvester, which exerts in return an external load force $F_{L}$ on the mechanism’s output. This effect can be represented by a stiffness $K_{H}$, connected in series with the mechanism’s stiffness $K_{Mech}$ [28]. $F_{L}$ can therefore be related to $Y_{ex}$ as:

(6) $$F_{L}=\frac{Y_{ex}}{K_{H}^{-1}+K_{Mech}^{-1}}.$$

Using $X_{in}$, $Y_{ex}$, and $Y_{L}$, from (3)–(5), the displacement amplification ratio (AR) in (2) can be calculated. The external reactions $A_{x},A_{y},A_{M}$ at the supporting end “A” and the internal reactions $B_{x}$, $B_{M}$ at “B” are determined from the following equilibrium Equations (7)–(9), and the boundary condition Equations (10) and (11):

(7) $$\begin{array}{ccc} \sum F_{x} & =0\; & A_{x}+B_{x}+F_{in}=0, \end{array}$$

(8) $$\begin{array}{ccc} \sum F_{y} & =0\; & A_{y}-\frac{F_{L}}{2}=0, \end{array}$$

(9) $$\begin{array}{ccc} \sum M_{A} & =0\; & A_{M}+B_{M}+B_{x}\;\left(l_{1}+l_{2}+l_{3}\right)\\ & \; & -\frac{F_{L}}{2}\;\left(\left(l_{4}+l_{5}+l_{6}\right)\;\cos\theta_{5}+l_{7}\right)\\ & \; & -B_{x}\;\left(l_{4}+l_{5}+l_{6}\right)\;\sin\theta_{5}\\ & \; & +F_{in}\left(l_{1}+l_{2}\right)=0, \end{array}$$

where $\theta_{5}$ is the inclination angle of the fifth segment, shown in Figure 3.

Using Castigliano’s energy theorem and due to the mechanism’s symmetry, both the horizontal displacement ($u_{B_{x}}$) and the rotation at the 7th segment ($\theta_{7}$) vanish:

(10) $$\begin{array}{cc} u_{B_{x}}= & \sum_{i=1}^{7}\;\int_{0}^{l_{i}}\frac{M_{i}\;\frac{d\;M_{i}}{d\;B_{x}}}{E\;I_{i}}\;ds=0, \end{array}$$

(11) $$\begin{array}{cc} \theta_{7}= & \sum_{i=1}^{7}\;\int_{0}^{l_{i}}\frac{M_{i}\;\frac{d\;M_{i}}{d\;B_{M}}}{E\;I_{i}}\;ds=0, \end{array}$$

where $M_{i}$ is the internal reaction moment for each mechanism segment *i*, represented from statics as:

$$\begin{array}{cc} M_{1}= & A_{M}+A_{x}\;s_{i},\\ M_{2}= & A_{M}+A_{x}\;\left(l_{1}+s_{i}\right),\\ M_{3}= & A_{M}+A_{x}\;\left(l_{1}+l_{2}+s_{i}\right)+F_{in}\;s_{i},\\ M_{4}= & A_{M}+A_{x}\;\left(l_{1}+l_{2}+l_{3}-s_{i}\;\sin\left(\theta_{5}\right)\right)\\ & +F_{in}\;\left(l_{3}-s_{i}\;\sin\left(\theta_{5}\right)\right)+\frac{F_{L}}{2}\;s_{i}\;\cos\left(\theta_{5}\right)),\\ M_{5}= & A_{M}+A_{x}\;\left(l_{1}+l_{2}+l_{3}-\left(l_{4}+s_{i}\right)\;\sin\left(\theta_{5}\right)\right)\\ & +F_{in}\;\left(l_{3}-\left(l_{4}+s_{i}\right)\;\sin\left(\theta_{5}\right)\right)\\ & +\frac{F_{L}}{2}\;\left(l_{4}+s_{i}\right)\;\cos\left(\theta_{5}\right)),\\ M_{6}= & A_{M}+A_{x}\;\left(l_{1}+l_{2}+l_{3}-\left(l_{4}+l_{5}+s_{i}\right)\;\sin\left(\theta_{5}\right)\right)\\ & +F_{in}\;\left(l_{3}-\left(l_{4}+l_{5}+s_{i}\right)\;\sin\left(\theta_{5}\right)\right)\\ & +\frac{F_{L}}{2}\;\left(l_{4}+l_{5}+s_{i}\right)\;\cos\left(\theta_{5}\right)),\\ M_{7}= & A_{M}+A_{x}\;\left(l_{1}+l_{2}+l_{3}-\left(l_{4}+l_{5}+l_{6}\right)\;\sin\left(\theta_{5}\right)\right)\\ & +F_{in}\;\left(l_{3}-\left(l_{4}+l_{5}+l_{6}\right)\;\sin\left(\theta_{5}\right)\right)\\ & +\frac{F_{L}}{2}\;\left(l_{4}+l_{5}+l_{6}+s_{i}\right)\;\cos\left(\theta_{5}\right)). \end{array}$$

The second moments of area for segments 2, 3, 5, and 7 are equal (i.e., $I_{2}=I_{3}=I_{5}=I_{7}$). For Segment 1, the right circular hinge is divided into subsegments $rc_{1}$ and $rc_{2}$ (see Figure 3). Their second moments of areas $I_{rc_{1}}$, and $I_{rc_{2}}$ are expressed as [15]:

$$\begin{array}{cc} I_{rc1}= & \frac{w}{12}\;{\left(t_{rc}+2\;\left(r_{rc}-\sqrt{r_{rc}^{2}-s_{rc1}^{2}}\right)\right)}^{3},\\ I_{rc2}= & \frac{w}{12}\;{\left(t_{rc}+2\;\left(r_{rc}-\sqrt{r_{rc}^{2}-{\left(r_{rc}-s_{rc2}\right)}^{2}}\right)\right)}^{3}, \end{array}$$

where *w* is the width of the mechanism, and $t_{rc}$ and $r_{rc}$ are the joints thickness and radius of curvature, respectively, as shown in Figure 1. Segments 4 and 6, which are bounded by corner filleted hinges, are further divided into three subsegments, $cf_{1}$, $cf_{2}$ and $cf_{3}$, as shown in Figure 3. The second moments of areas $I_{cf_{1}}$, $I_{cf_{2}}$, and $I_{cf_{3}}$ are expressed as [15]:

$$\begin{array}{cc} I_{cf1}= & \frac{w}{12}\;{\left(t_{cf}+2\;\left(r_{cf}-\sqrt{r_{cf}^{2}-s_{cf1}^{2}}\right)\right)}^{3}\\ I_{cf2}= & \frac{w}{12}\;t_{cf}^{3}\\ I_{cf3}= & \frac{w}{12}\;{\left(t_{cf}+2\;\left(r_{cf}-\sqrt{r_{cf}^{2}-{\left(r_{cf}-s_{cf3}\right)}^{2}}\right)\right)}^{3} \end{array}$$

where $t_{cf}$, $l_{cf}$, and $r_{cf}$ represent the joint’s thickness, length, and radius of curvature, respectively, as shown in Figure 1.

The stresses in the mechanism are not allowed to exceed its material yield strength and endurance limit. For low-frequency oscillatory excitation, the effect of fatigue is thus ignored. The normal stress $\sigma_{n}$ at any Segment *i* within the mechanism is primarily caused by the bending moment $M_{i}$ and the axial load $N_{i}$, and can be expressed as:

(12) $$\sigma_{n}=\frac{M_{i}}{S_{i}}+\frac{N_{i}}{A_{i}},$$

where $A_{i}$ is the cross-sectional area, and $S_{i}$ is the section modulus, defined as the ratio between the second moment of area $I_{i}$ and the distance from the neutral axis to the outer surface. The normal forces $N_{i}$ at the different mechanism segments are expressed in Table 1.

The displacement amplification and the stresses were also evaluated numerically by finite element methods, using a finite element (FE) software package. The model was meshed using tetrahedral and triangular elements of average, and minimum element qualities of 0.6822, and 0.1329, respectively.

## 3. Experimental Validation

**Figure 4:**
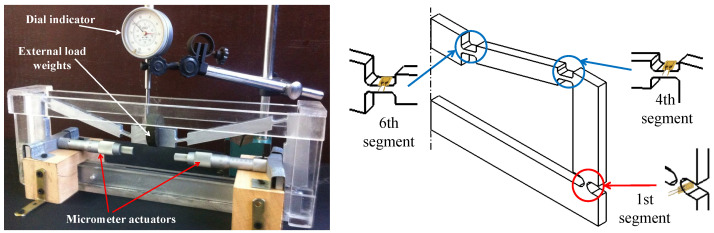
Experimental validation setup showing the locations of strain gauges on the mechanism.

The analytical and finite element models were both verified experimentally by constructing the mechanism with the dimensions presented in Table 2. The mechanism was cut out of an 8 mm-thick Poly(methyl methacrylate) (PMMA) sheet, using a CO_2_ laser cutting machine. The material has a Young’s modulus (*E*) of 3.2 GPa, and Poisson’s ratio (ν) of 0.327. The input displacements $X_{in}$ were provided using two micrometer actuators, with 0.05 mm accuracy, applied in the opposite directions. $X_{in}$ was varied between 0.1 mm and 0.5 mm (at 0.1 mm increments), while the external load $F_{L}$ was applied to the top of the mechanism using weights that were varied between 1 N to 5 N (at 1 N increments). A dial indicator, with an accuracy of 0.01 mm, was used to measure the output displacements. Figure 4 shows the setup for the experimental validation. Six strain gauges were fixed at the middle of each flexure hinge (Segments 1, 4, and 6), at the locations shown in Figure 4. These locations were selected for being the mechanism’s weakest segments. The strain gauges were connected to strain meters (DP25B-S, Omega, CT, USA), which were calibrated to provide the average strain.

**Figure 5:**
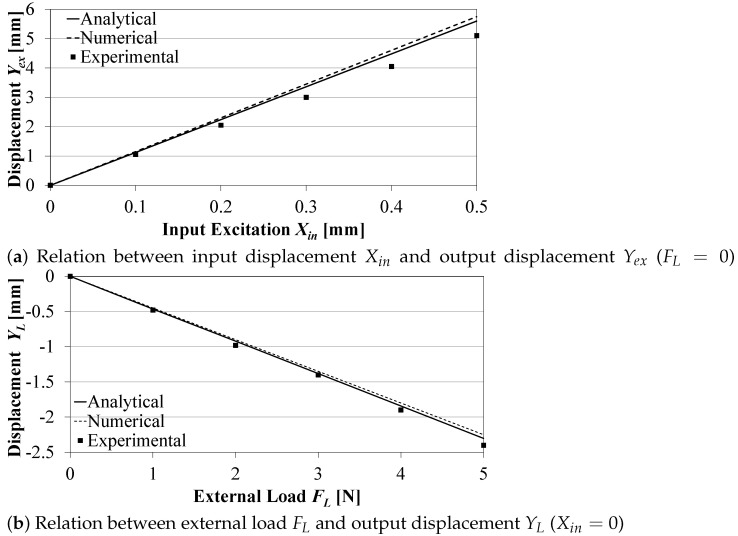
Effect of the input displacement $X_{in}$ and the external load $F_{L}$ on the output displacement.

The output displacements $Y_{ex}$, at no external load, and $Y_{L}$, at no input displacement, are determined from Equations (4) and (5), respectively. Figure 5a,b show the effect of the input displacement $X_{in}$ on $Y_{ex}$ and the effect of the load $F_{L}$ on $Y_{L}$, respectively, as predicted analytically, numerically, and as measured experimentally. The negative sign of $Y_{L}$ indicates its downwards direction. Both Figure 5a,b show good agreement between the analytical, numerical, and experimental results with a maximum error less than 9%, which validates the proposed analytical model for $X_{in}$.

**Figure 6:**
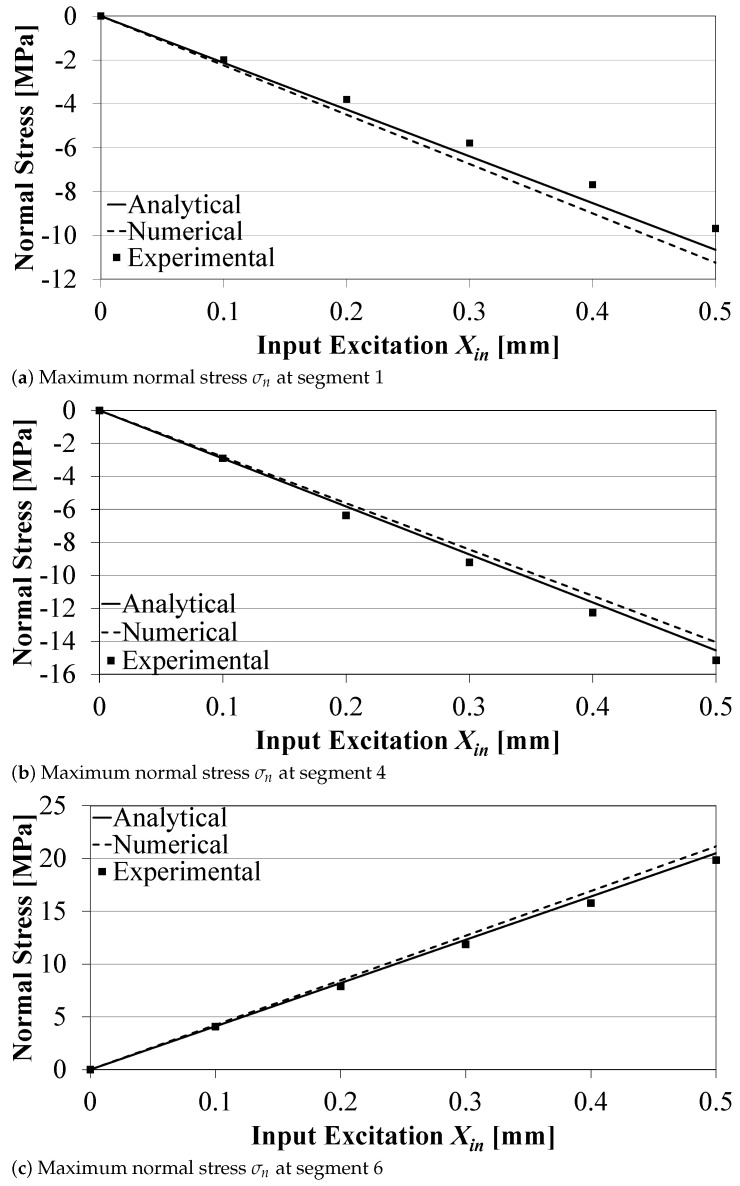
Normal stresses on the compliant mechanism due to $X_{in}$ only ($F_{L}=0$).

**Figure 7:**
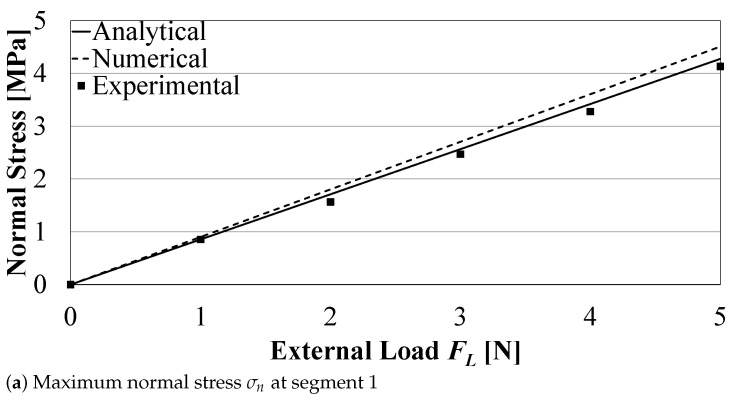

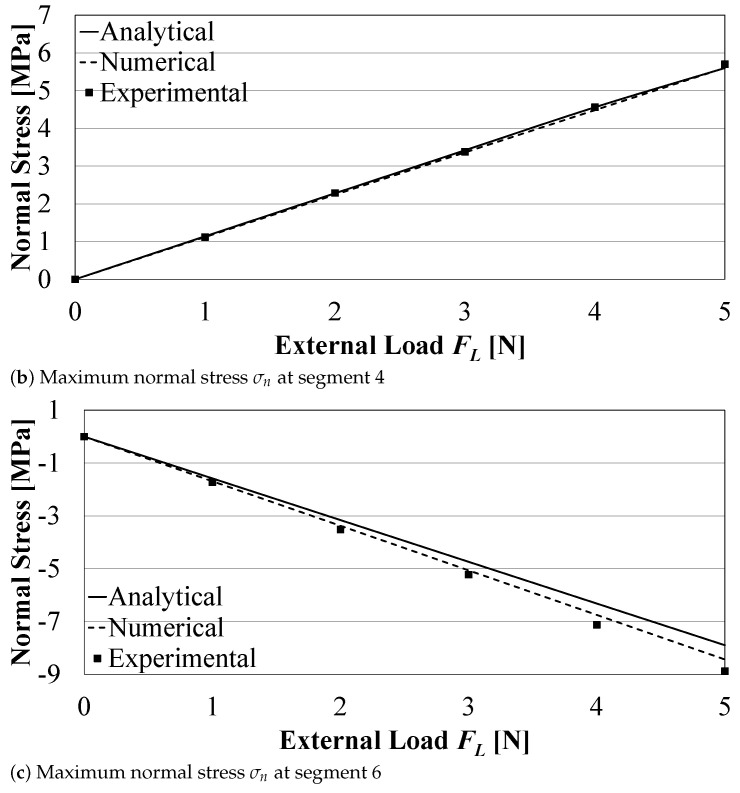
Normal stresses on the compliant mechanism due to $F_{L}$ only ($X_{in}=0$).

The maximum normal stress $\sigma_{n}$ at the middle section of the right circular hinge (Segment 1) was evaluated analytically, using Equation (12), numerically, and experimentally with the stresses estimated from the measured strains using Hooke’s law. Two cases were investigated: (1) $\sigma_{n}$ caused by $X_{in}$ only (at $F_{L}=0$), and (2) $\sigma_{n}$ caused by $F_{L}$ only (at $X_{in}=0$), as shown in Figure 6a and Figure 7a, respectively. Similarly, Figure 6b,c, and Figure 7b,c show the maximum stresses developed at the middle sections of the two upper flexure hinges (Segments 4 and 6). Figure 6 and Figure 7 show good agreement between the analytical and numerical model results and those from the experiments, for different $X_{in}$ and $F_{L}$. Errors between analytical, numerical, and experimental are found to be less than 10% at large excitations, and/or large loads, which can be attributed to calculation assumptions, change in configuration angles at large deformations, in addition to experimental measurement errors. Results indicate that the analytical model can be used to calculate the normal stresses in flexure joints.

## 4. Energy Harvesting Using a Unimorph Piezoelectric Cantilever

**Figure 8:**
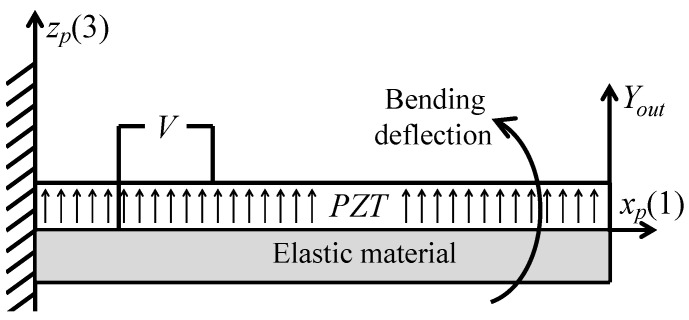
Schematic of the unimorph piezoelectric beam.

A unimorph piezoelectric cantilever was integrated with the symmetric five-bar compliant mechanism to harvest energy from the amplified displacement. Figure 8 shows a schematic drawing of the used unimorph piezoelectric beam. The beam is fixed from one side with its free tip connected to the excitation source. Here, the harvester’s excitation source is the amplified displacement $Y_{out}$. The excitation causes bending deformation in the piezoelectric beam, which generates a voltage across the piezoelectric layers. The poles of the piezoelectric material are oriented in the $z_{p}$-axis direction $z_{p}\left(3\right)$; thus, electric voltage is generated between piezoelectric upper and lower layers. The generated bending stresses are in the $x_{p}\left(1\right)$ direction, as shown in Figure 8. Thus, the piezoelectric constant used in the calculations is $d_{31}$.

The constitutive equation for the unimorph piezoelectric bender with a fixed-free boundary condition is expressed as [28]:

(13) $$Y_{out}=a_{11}\;F_{L}+a_{12}\;V.$$

where $Y_{out}$ is the tip deflection of the piezoelectric beam, $F_{L}$ and *V* are the force and the voltage of the piezoelectric beam, respectively. For equal lengths of piezoelectric layer and the elastic layer, the coefficients $a_{11}$ and $a_{12}$ are expressed by:

(14) $$\begin{array}{cc} a_{11}= & \frac{4\;{L_{b}}^{3}\;}{E_{P}w_{b}\;t_{m}^{3}}\;\frac{\left(1+\eta \;\xi^{3}\right)}{(\eta^{3}\;\xi^{4}+\;\eta^{2}\left(\;4\xi^{3}+6\;\;\xi^{2}+4\;\;\xi \right)+\eta )} \end{array}$$

(15) $$\begin{array}{cc} a_{12}= & \frac{d_{31}\;{L_{b}}^{2}}{t_{m}^{2}}\;\frac{6\;\eta \;\xi^{3}}{\left(\eta^{2}\;\xi^{4}+4\;\eta \;\xi^{3}+6\;\eta \;\xi^{2}+4\;\eta \;\xi +1\right)}\; \end{array}$$

where $L_{b}$ is the beam length, $E_{P}$ is the Young’s modulus of the piezoelectric material, $w_{b}$ is the beam width, $t_{m}$ is the thickness of elastic layer, η is the material modulus of elasticity ratio for the piezoelectric beam ($\eta =(E_{m}/E_{P})$), $E_{m}$ is the Young’s modulus of the nonpiezoelectric material, ξ is the thickness ratio for piezoelectric beam ($\xi =(t_{m}/t_{p})$), $t_{p}$ is the thickness of the piezoelectric layer, and $d_{31}$ is the piezoelectric constant.

**Figure 9:**
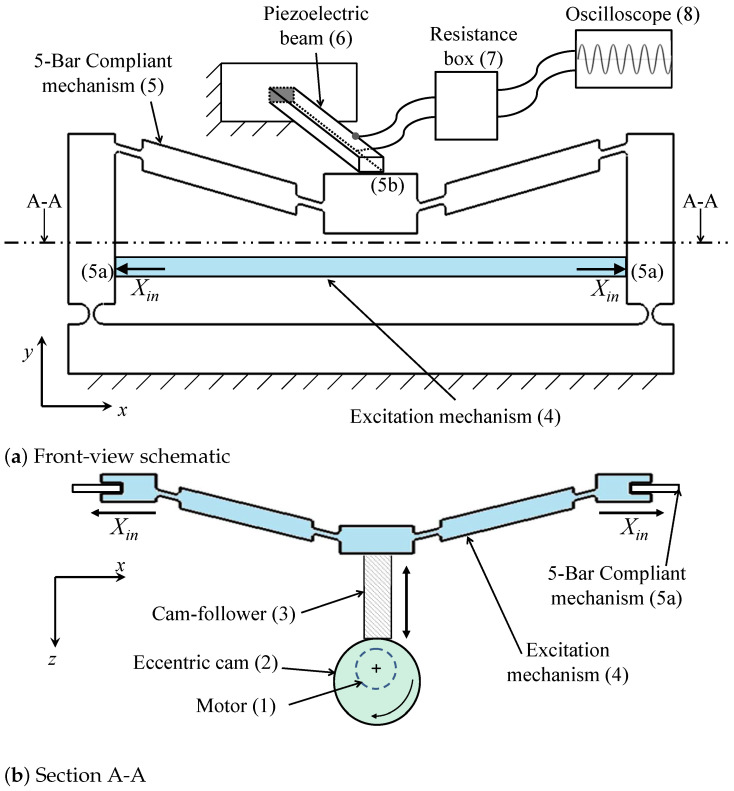
Schematic of experimental setup for energy harvesting.

**Figure 10:**
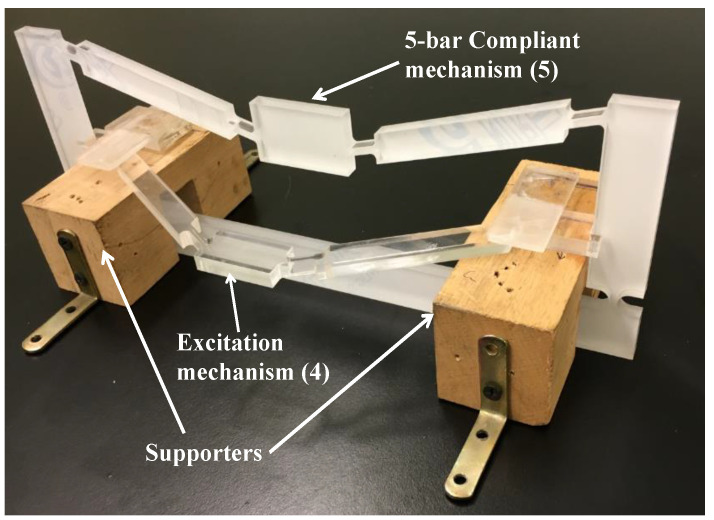
A photograph of the exciter mechanism connected to amplification displacement mechanism.

Figure 9a,b shows schematics of the experimental setup. A DC motor (1) (24-Volts, 230-Watts) drives an eccentric circular cam (2) that produces a reciprocating motion in a cam-follower (3). The cam-follower (3) pushes against an excitation mechanism (4), which is a symmetric three-bar compliant mechanism that transmits the reciprocating follower motion to the inputs of the 5-bar compliant mechanism (5a) ($X_{in}$). The output of the mechanism (5b) moves with an amplified displacement $Y_{out}$ and pushes against the free end of the piezoelectric beam (6). The terminals of the piezoelectric beam are electrically connected to a resistance box (7) where the resistance load is controlled. The resistance box (7) is connected to a digital oscilloscope (8) for voltage measurement and data storage. Figure 10 shows a photograph of the three-bar mechanism connected to the five-bar displacement amplification mechanism.

The properties and dimensions of the piezoelectric beam are listed in Table 3. The used unimorph stripe is (#40-2030, APC Int Ltd., Mackeyville, PA, USA). The coefficient $a_{11}$ was calculated using (14) and found to be 6.19 mm/N, which represents the compliance in the beam. The coefficient $a_{12}$ was calculated using (15) as 8.78 mm/V.

The DC motor was controlled to run at a low angular speed of 0.75 Hz. The cam-excitation mechanism assembly provided the compliant mechanism with an input excitation displacement $X_{in}$ of 0.16 mm. A resistance box was used to change the resistance between 0 and 7 MΩ. The peak voltage value was recorded at every used resistance using a digital oscilloscope (TBS1064, Tektronix, OR, USA).

**Figure 11:**
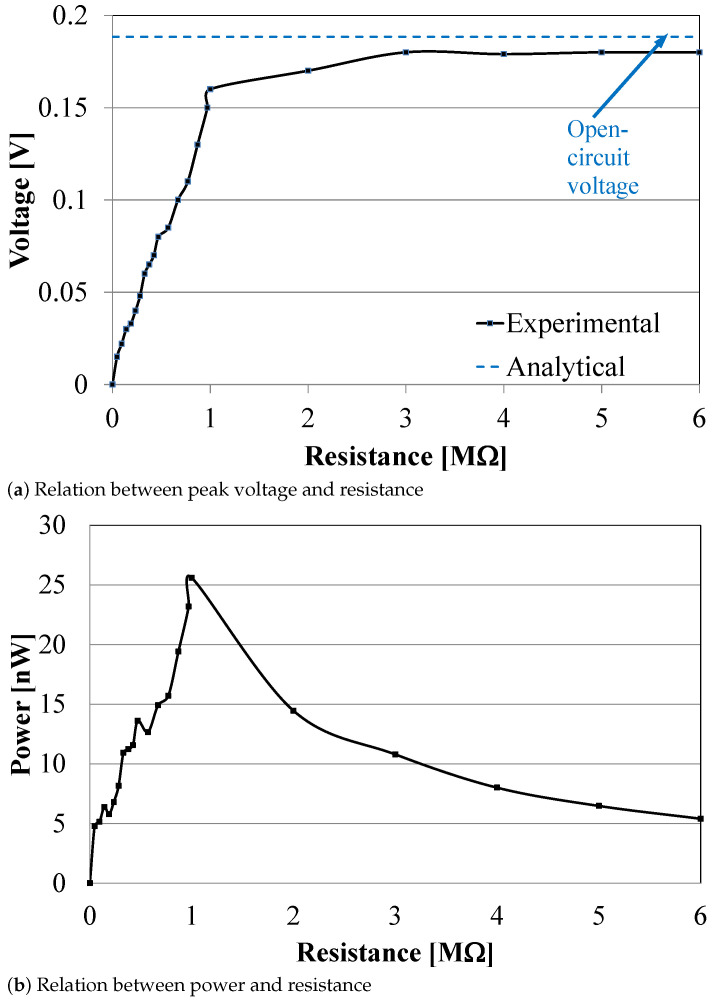
Experimental harvested voltage and power.

The relation between the output peak voltage and the resistance is presented in Figure 11a. The figure shows that the output voltage changes with the change in load; however, it saturates at a maximum value of 0.18 V for resistances larger than 3 MΩ.

At the same input excitation of $X_{in}=0.16$ mm, Equations (1), and (4)–(6) were used to calculate the corresponding $Y_{ex}$, $F_{L}$, $Y_{L}$, and $Y_{out}$. The open-circuit voltage was thus calculated analytically using Equations (13)–(15) and was found to be 0.1884 Volts, as shown in Table 4. Inspection of Figure 11a reveals that the experimental voltage converges to the open-circuit value as the resistance increases, as expected from the piezoelectric generator. For resistances larger than 3 MΩ, the error in voltage was found to be 4.7%, which validates the analytical model for the piezoelectric beam, thus it can be used for future design and testing of harvesting systems.

The harvested power can be calculated using the equation:

(16) $$Power=V^{2}/R,$$

where *V* is voltage, and *R* is the load resistance. Figure 11b shows the effect of the load resistance on the output power. The maximum power harvested for the setup was 25.6 n*W* at 1 MΩ resistance. Without the displacement amplification mechanism, the maximum harvested power would have been 0.33 n*W* at an open-circuit voltage of 0.0182 Volts, as calculated analytically using Equations (13)–(15).

## 5. Conclusions

In this paper, an analytical method using Castigliano’s energy theorem was formulated for evaluating the output displacement and normal stresses for a symmetric five-bar compliant mechanism that is driven by an input displacement. Determination of the output displacement and amplification ratio includes the effects of both the input excitation displacement and the external load. This presents an attempt to study the compliant mechanism as an energy harvester that derives its input from a persistent motion and amplifies it to drive a generator. The analytical model can be used to optimize the mechanism dimensions and design parameters for both maximized harvested power and amplification ratios. The analytical model can also be used to estimate the normal stresses in the flexure joints, which is particularly interesting when designing mechanisms of maximum amplification ratios, but with normal stresses that are lower than the material’s strength thresholds. The effect of fatigue can be disregarded for low-frequency oscillatory excitation as long as the material’s endurance limit is not exceeded. The analytical and numerical results were validated experimentally.

When integrating the mathematical model of the mechanism with another representing the piezoelectric energy harvester, the open-circuit voltage was calculated. This voltage was found to be in agreement with that achieved experimentally using a unimorph piezoelectric cantilever operating at low-frequency harmonic excitation inputs. With the proposed mechanism, the piezoelectric generator’s open-circuit voltage equals the amplification ratio (AR) times that without the amplification mechanism. Accordingly, the generated power at a load equals the square of the amplification ratio (${AR}^{2}$) times that without amplification. The results validate the proposed models and confirm the potential of using displacement amplification compliant mechanisms in vibration-based energy harvesting applications.

## Figures and Tables

**Table 1:** Normal force $N_{i}$ in each segment *i*.

Segments 1, 2, and 3	$N_{i}=-\frac{F_{L}}{2}$
Segments 4, 5, and 6	$N_{i}=A_{x}\;\cos\;\theta_{i}+\frac{F_{L}}{2}\;\sin\;\theta_{i}$
Segment 7	$N_{i}=A_{x}$

**Table 2:** Mechanism dimensions (mm).

Hinges	$t_{rc}$	$r_{rc}$	$t_{cf}$	$r_{cf}$	$l_{cf}$
	3.75	5	3.75	2	15
Links	$l_{1}$	$l_{2}$	$l_{3}$	$l_{4}$	$l_{5}$
	10	85	15	85.5	15
Others		*w*		$\theta_{5}$	
		8		15°	

**Table 3:** Piezoelectric beam dimensions and properties.

Material	$d_{31}$				$E_{P}$				$E_{m}$			
	125 × 10^−9^ mm/V				80 GPa				200 GPa			
Dimensions	$t_{P}$			$t_{m}$			$L_{b}$			$w_{b}$		
	0.24 mm			0.16 mm			33 mm			2 mm		

**Table 4:** System parameter calculations.

Parameter	*X_in_* (mm)	*Y_ex_* (mm)	*F_L_* (N)	*Y_L_* (mm)	*Y_out_* (mm)	*AR*	*V* (V)
Value	0.16	1.787	0.289	0.133	1.654	10.33	0.1884
Equation		(4)	(6)	(5)	(1)	(2)	(13)–(15)

## Data Availability

Data sharing is not applicable to this article.

## Abbreviations

The following abbreviations are used in this manuscript:

*AR*	Amplification ratio
PMMA	Poly(methyl methacrylate)
PRBM	Pseudo-rigid-body models
PZT	Lead Zirconate Titanate (piezoelectric ceramic material)
